# The Effect of UV Treatment on the Osteoconductive Capacity of Zirconia-Based Materials

**DOI:** 10.3390/ma9120958

**Published:** 2016-11-24

**Authors:** Miha Brezavšček, Ahmed Fawzy, Maria Bächle, Taskin Tuna, Jens Fischer, Wael Att

**Affiliations:** 1 Department of Prosthodontics, School of Dentistry, Albert-Ludwigs University, Hugstetter Strasse 55, 79106 Freiburg, Germany; miha_brezavscek@yahoo.com (M.Br.); ahmed.fawzy@uniklinik-freiburg.de (A.F.); maria.baechle-haas@uniklinik-freiburg.de (M.Bä.); ttuna@ukaachen.de (T.T.); 2 Institute for Dental Materials and Engineering, University Hospital for Dental Medicine, University of Basel, 4056 Basel, Switzerland; Jens.Fischer@uzb.ch

**Keywords:** osseointegration, zirconia, implant surface, photofunctionalization, ultraviolet treatment, bone

## Abstract

Objective: Improvements in the bioactivity of zirconia implants for accelerated healing and reduced morbidity have been of continuing interest in the fields of dentistry and orthopedic surgery. The aim of the present study was to examine whether UV treatment increases the osteoconductivity of zirconia-based materials. Materials and Methods: Smooth and rough zirconia-based disks and cylindrical implants were treated with UV light for 15 min and subsequently placed in rat femurs. Surface characterization was performed using scanning electron microscopy (SEM), atomic force microscopy (AFM), X-ray photoelectron spectroscopy (XPS) and contact angle measurements. Results: In vivo histomorphometry revealed that the percentage of bone-implant contact and the amount of bone volume, formed around UV-treated implants, increased by 3–7-fold for smooth surfaces and by 1.4–1.7-fold for rough surfaces compared to non-treated specimens at Weeks 2 and 4 of healing, respectively. A biomechanical test showed that UV treatment accelerated the establishment of bone-zirconia integration and enhanced the strength of the bone-implant interface by two-fold. Additionally, surface characterization of the zirconia disks revealed that UV treatment decreased the amount of surface carbon and converted the hydrophilic status from hydrophobic to superhydrophilic. Conclusions: This study indicates that UV light pretreatment enhances the osteoconductive capacity of zirconia-based materials.

## 1. Introduction

Biomedical zirconia was introduced in 1969 into medicine to solve the problem of alumina brittleness in hip replacement procedures and has since been used for various joint replacement appliances in orthopedic surgery [1,2]. Today, the most frequently-studied material is yttria-stabilized zirconia, which is also known as tetragonal zirconia polycrystal (TZP) [3]. These Y-TZP materials contain 2%–3% mol yttrium oxide (Y_2_O_3_) and are composed entirely of tetragonal grains with diameters on the order of hundreds of nanometers. The tetragonal fraction retained at room temperature and, subsequently, the material’s mechanical properties are dependent on grain size, yttrium oxide content and the degree of constraint exerted on them by the matrix [4]. Y-TZP presents various interesting characteristics, such as low porosity, high density and high bending and compression strength, proving that it is suitable for biomedical application. These mechanical properties, as well as its bright color have contributed to the popularity of zirconia in the fields of prosthetic dentistry and dental implantology. In fact, many research groups recommend the use of zirconia implants in the aesthetic zone to avoid problems with titanium when the quantity of soft tissue is not sufficient to mask its gray color [5]. Currently, zirconia is considered to be one of the most frequently-used materials after titanium, especially in the dental field [3,6].

According to several animal studies, the osseointegration of zirconia implants was found to be similar to that of titanium implants [7,8,9,10,11]. Moreover, it has been demonstrated that modifying surface characteristics, such as topographical configuration and physicochemical properties, can influence the osteoconductivity of zirconia implants in a similar manner as has been reported for titanium implants [8,12,13,14]. Here, zirconia implants with roughened surfaces exhibit enhanced osteoconductive capacity, leading to improved osteogenesis and stronger biomechanical fixation when compared to smooth zirconia surfaces [14,15,16]. Nevertheless, the surface modification of zirconia is considered to be technically more demanding than for titanium. This is mainly due to the fact that conventional surface modification techniques employed for titanium either have no effect on zirconia or do not yield satisfactory surface roughness [6]. So far, the method of choice for increasing the surface roughness of zirconia has been air-borne particle abrasion [6,8]. However, it is highly possible that this surface modification technique may jeopardize the material’s resistance to low-thermal-degradation (LTD), leading to an increased risk of surface degradation that may subsequently result in premature failure of the implant material [3] Alternative techniques to air-borne particle abrasion, such as selective infiltration-etching (SIE), laser surface modification (CO_2_, Er:YAG, femtosecond laser) and chemical methods (sol-gel, alternate soaking process, bio-mimetic route), have previously been described [17,18,19,20,21,22]. Recently, a new method for zirconia surface modification that combines Al_2_O_3_ sandblasting together with acid etching and heat treatment has been documented [23]. However, these techniques are still at the experimental stage and have not been verified under in vivo conditions [17,18,21]. Clearly, there is an immediate need for new methods to increase the osteoconductive capacity of zirconia-based implant surfaces without jeopardizing their mechanical properties.

Recently, the effect of ultraviolet (UV) light on titanium surfaces proved to enhance their bioactivity and osteoconductive capacity by promoting the osteogenic cell attachment and proliferation, as well as the protein adsorption. This phenomenon, termed ‘UV light-mediated photofunctionalization’, has been found to be associated with the photochemical and photocatalytic removal of hydrocarbons from the titanium surfaces, the alteration of the surface electrostatic properties and the generation of hydrophilicity [24,25,26]. It was found that the minimum UV light energy required to induce photo-catalytic activity to excite an electron from the valence band to the conduction band of the TiO_2_ must exceed 3.2 eV, which corresponds approximately to a 370-nm wavelength of UV light, referred to as UVA. On the other hand, the direct decomposition of hydrocarbon by UVC light in the lower range at around a 250-nm wavelength is also required [27,28,29,30].

Compared to untreated surfaces, in vivo and in vitro evaluations demonstrated increased osteoblast activity, enhanced bone formation and improved bone-implant anchorage on UV-pretreated titanium surfaces [26,30,31].

The effect of UV light treatment was also tested on smooth zirconia surfaces [28]. In a similar manner to titanium surfaces, UV-treated zirconia surfaces exhibited an enhanced osteoblast response, which was characterized by an accelerated and augmented cell attachment, accelerated cell spread and cytoskeletal development with increased proliferation [32]. Such amplification in osteoblast activity resulted in a two-fold increase of ALP-positive areas at an initial stage and in a doubling of the area of mineralization in their mature stage [33]. These findings indicated that UV irradiation might provide a novel approach to develop more bioactive zirconia implants. Based on the previous in vitro findings, an evaluation of UV-treated zirconia implants placed in bone tissue would provide valuable information about their osteoconductivity and possible clinical application. Therefore, the aim of this study was to examine the effect of UV light treatment on the osteoconductive capacity of zirconia surfaces in an experimental animal model.

## 2. Model and Theory

### 2.1. Zirconia Samples, UV Light Treatment and Surface Analyses

**Figure 1:**
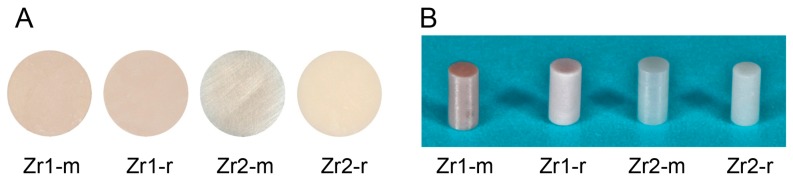
(**A**) Zirconia disks and (**B**) cylindrical rods.

Disks (diameter, 20 mm; thickness, 1.5 mm) (Figure 1A) and cylindrical rods (diameter, 1 mm; length, 2 mm) (Figure 1B) were prepared from two zirconia-based materials. The material composition of Zr1 and Zr2 is described elsewhere [25]. In brief, material Zr1 consisted of the metal oxides ZrO_2_ (85.7 wt%), Al_2_O_3_ (8.3 wt%), Y_2_O_3_ (4.3 wt%) and La_2_O_3_ (1.7 wt%). Zr2 represented a more conventional yttrium tetragonally-stabilized zirconium oxide containing 93 wt% ZrO_2_, 5 wt% Y_2_O_3_, 1.9 wt% HfO_2_ and 0.1 wt% Al_2_O_3_. (Vita Zahnfabrik, Bad Säckingen, Germany). While the smooth surface of material Zr1 was just the as-sintered material, the smooth surface of Zr2 was additionally polished with 3-μm diamond paste, whereas roughened surfaces (Zr1-r, Zr2-r) were prepared by sandblasting with Al_2_O_3_ (grain size 105 μm, pressure 6 bar), followed by acid etching using 38%–40% hydrofluoric acid (HF) and a heat treatment at >900 °C, in order to smoothen the sharp edges resulting from the etching procedure (Patent Number US 8,257,606 B2). All specimens were cleaned with double distilled water, followed by ultrasonic cleaning in double distilled water for 5 min and air-drying. Subsequently, the specimens were sterilized in a low-temperature hydrogen peroxide gas plasma sterilizer at 55 °C (STERRAD^®^, 100NX™ System, Johnson & Johnson Medical, Norderstedt, Germany), sealed and stored for one month in the dark (temperature, 23 °C; humidity, 60%). Half of the samples from each group were treated with UV light for 15 min using a UV activation device (TheraBeam, Affiny, Ushio Inc., Otemachi, Japan). The UV light was delivered as a spectral mixture via a single source at λ = 360 nm and λ = 250 nm. The disk specimens were used for the evaluation of physical and chemical surface properties, whereas the cylindrical rods were employed as implant analogs for in vivo assessment.

#### 2.1.1. Surface Topographic Feature Analysis

The surface morphology of untreated and UV-treated samples was examined by scanning electron microscopy (SEM) with a LEO 1525 Field Emission Gun (FEG SEM, Zeiss, Jena, Germany). The specimens were coated with a thin layer of electron conductive fast-drying silver suspension (Silver Print, Provac AG, Liechtenstein) at the margins before scanning at various magnifications (20×/50×/100×/500×/5000×/30,000×). To quantify the topographic features, a laser profilometer (UBM Microfocus), a tactile profilometer (Hommel Etamic, Turbowave, VS-Schwenningen, Germany) and atomic force microscopy (AFM) with a Nanoscope IIIa (Veeco-Digital Instruments, Santa Barbara, CA, USA) were employed. The measuring lengths of the tactile and the optical profilometer were 4.8 mm and 0.5 mm, respectively. The AFM had a scan surface area of 30 µm × 30 µm and a 2-µm z-range, which was considered to be representative for the evaluation of the surface roughness of each specimen. The standard descriptors of surface roughness, such as arithmetic roughness (Ra), root mean square roughness (Rms), mean peak-to-valley height (Rz) and the surface area, were calculated.

#### 2.1.2. Surface Crystalline Property Analysis

The crystalline structure of the samples was determined by an X-ray diffractometer (XRD) equipped with a Cu-Kα-type X-ray source (D 8 Advance X-ray diffractometer, Bruker AXS, Karlsruhe, Germany). The identification and correction of peaks (monoclinic/tetragonal/cubic) was based on the ICSD (Inorganic Crystal Structure Database) database (http://www2.fiz-karlsruhe.de/icsd_home.html).

#### 2.1.3. Evaluation of the Hydrophilic Status

The hydrophilic property of different zirconia disks was examined by measuring the contact angle of a 1-μL H_2_O droplet using an automated contact angle meter (Dataphysics C.A.S.O.C.A., Model OCA 10, Data-Physics Instruments, Filderstadt, Germany). The H_2_O contact angles at four different areas on the zirconia surface were averaged. All procedures were performed in a Class 10 clean room under controlled conditions of 20 °C and 46% humidity.

#### 2.1.4. Examination of Surface Elemental Composition

The chemical composition of zirconia surfaces was evaluated by electron spectroscopy for chemical analysis (ESCA). ESCA was performed by X-ray photoelectron spectroscopy (XPS) (Perkin Elmer PHI 5600 ESCA System, Physical Electronics, Inc., Chanhassen, MN, USA) under high vacuum conditions (5 × 10^−8^ mbar). The system was used with a magnesium Ka X-ray source (X-ray voltage: 13 KV, Anode Power 300 W) at a take-off angle of 45°.

### 2.2. Surgery

Eighty-eight eight-week-old male Sprague-Dawley rats were used for the study. The rats were anesthetized by inhalation of 1%–2% isoflurane. After the legs were shaved and scrubbed with a 10% povidone-iodine solution, the distal aspect of the left and right femurs were carefully exposed via skin incision and muscle dissection. The flat surface of the distal femur was selected for implant placement. The implant site was prepared 11 mm from the distal edge of the femur by drilling first with a 0.8-mm round bur and enlarging it using reamers (#ISO 090 and 100). Profuse irrigation with sterile isotonic saline solution was used for cooling and cleaning. The implants were subsequently placed into the osteotomy and carefully pushed into place until the end of the implant was aligned with the femoral bone surface. After the implants were correctly positioned, the tissues were closed in layers. Muscle and skin were sutured separately with resorbable sutures (Vicryl, Ethicon GmbH, Norderstedt, Germany).

Eighty-eight rats were randomly allocated into two groups, 48 rats for the push-in test and 40 rats for histomorphometric analysis. The animals were further divided into four groups based on the type, as well as the surface topography of the zirconia samples for histomorphometric evaluation (10 rats each) and four groups for the push-in test (12 rats each). In all groups, each rat received one UV-treated implant in the left femur and one untreated implant in the right femur. In each group, animals were sacrificed at Week 2 and Week 4 after implant placement.

This study protocol has been approved by the Freiburg Animal Research Committee (Approval No. 35-0185.81/G-11/17).

### 2.3. Histological Processing

The animals were sacrificed with an overdose of CO_2_. The femurs were dissected free, and the bone segments containing the implants were harvested. After rinsing with saline, the specimens were immersed in 10% buffered formalin. Subsequently, the specimens were dehydrated in an ascending series of alcohol (Exakt Dehydration-system HS 501 digital, Ika-Labortechnik, Staufen, Germany) and finally embedded in photo-curing one-component resin (Technovit 9100 NEU, Heraeus Kulzer, Wehrheim, Germany). After polymerization of the resin, non-decalcified specimens were cut using a diamond saw and successively ground to a thickness of approximately 80–100 μm at the most upper part of the implant within the cortical bone with a grinding system (Exakt Apparatebau, Norderstedt, Germany). The specimens were then stained with toluidine blue (Fluka, Taufkirchen, Germany) and counterstained with Pararosanilin (Pararosanilin, Sigma-Aldrich, Deisenhofen, Germany). Subsequently, the specimens were rinsed with water and air dried.

### 2.4. Histomorphometry

Histological observations and computer-assisted histomorphometric analysis were performed at 10×, 20× and 40× magnification using an Axioscope (Zeiss, Jena, Germany) equipped with a color video camera SZH10 (Olympus, Hamburg, Germany) and evaluation software (Cell*, Olympus Soft Imaging Solutions GmbH, Münster, Germany). The histomorphometric analysis included the following parameters and corresponding equations:

Bone-implant contact (%) = (sum of the length of bone-implant contact)/(circumference of the implant) × 100.

The method of calculating of newly formed bone was previously described in several studies [30,34]. In detail, to calculate the bone to implant contact (BIC), fifteen lines were drawn parallel to the circumference of the implant at every 20 μm up to 300 μm from the implant surface. The length of the lines within the bone tissue was summed. The sum divided by the entire length of the line was defined as the bone rate (%). The data were plotted as line graphs to create the BIC; where the implant-bone contact was defined as the interface where bone tissue was located within 20 μm of the implant surface without any intervention of soft tissue.

Bone volume in the proximal zone (%) = (bone area in proximal zone)/(area of proximal zone) × 100.

The proximal zone is a circumferential zone within 50 μm of the implant surface.

### 2.5. Implant Biomechanical Push-In Test

**Figure 2:**
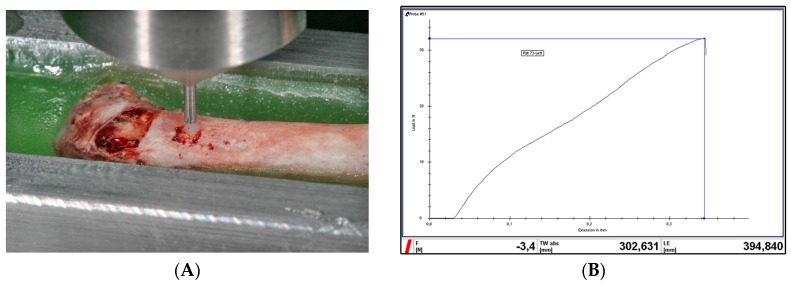
(**A**) Push in test: the implant is loaded in the universal testing machine; (**B**) a representative image showing the load-displacement curve during the push-in test. The highest load peak before a sudden drop in the curve denotes the push-in value.

The biomechanical strength of bone-implant integration was assessed via the implant biomechanical push-in test [35]. After sacrifice and dissection, femurs were immediately embedded in an autopolymerizing resin (Technovit 4071, Heraeus Kulzer, Wehrheim, Germany) using a custom-made metal mold. The implants were then loaded axially in a universal testing machine (Zwick Z010, Zwick GmbH & Co., KG, Ulm, Germany) using a 2000 N load cell and a 0.8 mm-diameter stainless-steel pushing rod with a crosshead speed of 1 mm/min (Figure 2A). The applied load and the displacement of the implant were monitored at a sampling rate of 4 Hz. The push-in value was determined by measuring the peak of the load-displacement curve (Figure 2B).

A statistical comparison between push-in values of non-treated and UV-treated implants was performed at each healing period (two and four weeks) and for each surface (smooth and rough).

### 2.6. Statistical Analyses

A linear (mixed) model was fitted to evaluate the effect of treatment (control, UV), surface (smooth, rough), material type (Zr1, Zr2) and healing time (14, 28 days) on the response variables (surface properties, BIC, push-in). From these models, least-square means were derived with 95% confidence intervals. Furthermore, relevant comparisons (of least square means) between treatment, material and time were carried out. Therefore, a Benjamini–Hochberg correction for multiple testing was applied (adjustment of *p*-values). Looking at histograms has checked the model assumptions, i.e., normal distribution of residuals, and normal probability plots. Differences of least-square (ls) means with corresponding 95% confidence intervals were plotted. All calculations were performed with the statistical software SAS 9.1.2 (SAS Institute Inc., Cary, NC, USA) using PROC MIXED.

## 3. Results

### 3.1. Topographic Features

**Figure 3:**
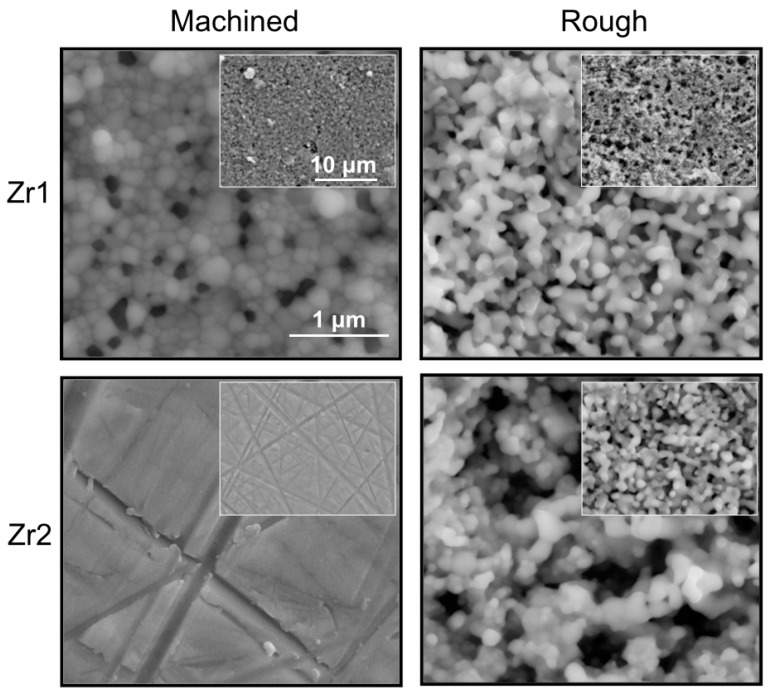
SEM images (30,000×) of Zr1-m, Zr1-r, Zr2-m and Zr2-r surfaces before UV treatment.

SEM images of Zr2-m revealed a smooth homogenous surface with superficial polishing patterns (Figure 3). On the other hand, Zr1-m exhibited a dense grainy structure, with well-defined grains of different sizes and contrast. The continuity of the surface was disrupted by homogenously distributed pores, which were similar in size to the well-defined grains, but distinct in their appearance (black spot-like appearance) (Figure 3). Furthermore, Zr1-r and Zr2-r exhibited a similar grainy structure, with deeper pits and lacunas. The form and the size of the grains were not clearly discernible, giving a coral-like appearance (Figure 3). UV treatment did not induce any changes in the topographic appearance of all tested samples.

The lowest surface roughness values were found in Zr2-m followed by Zr1-m, Zr1-r and Zr2-r (Table 1). The comparison of roughness values between untreated and UV-treated samples demonstrated that UV treatment did not significantly influence the surface roughness of different materials (Table 1).

### 3.2. Crystalline Properties of Zirconia Surfaces

**Figure 4:**
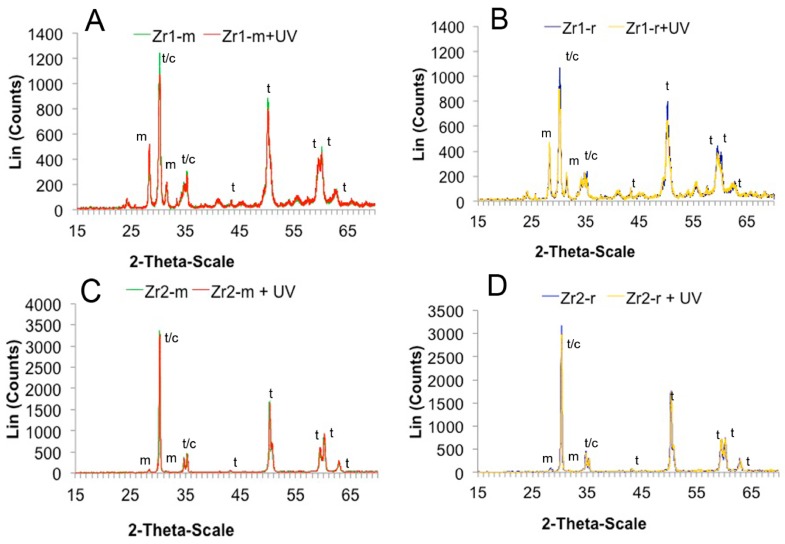
(**A**–**D**) UV light-induced changes in the crystalline structure of the zirconia specimens. X-ray diffraction (XRD) spectrum of the Zr1-m, Zr1-r, Zr2-m and Zr2-r surfaces used in the present study.

XRD analysis revealed a peak at 2Θ: 28.275° and 31.534° for all of the zirconia disks, which typically denotes the monoclinic phase. The samples also showed a peak at 2Θ: 30.3°, which is characteristic for the tetragonal-cubic phase of ZrO_2_ (Figure 4A–D). Furthermore, peaks specific for the tetragonal phase were identified.

Based on the XRD measurements, a distinct content of the crystalline phases was found between materials Zr1 and Zr2, as well as between different surfaces of the same material (Zr1-m vs. Zr1-r and Zr2-m vs. Zr2-r). The surface of material Zr2 contained a small percentage of the monoclinic phase (Zr2-m ≈ 3.4 at%, Zr2-r ≈ 4.9 at%), whereas a significantly higher monoclinic content was detected on surface of material Zr1 (Zr1-m ≈ 33.6 at%, Zr1-r ≈ 35 at%) (Figure 4A–D). After UV treatment was applied, a 19%–25% increase of the monoclinic phase was observed in material Zr1-m and Zr1-r (Zr1-m: ~40.1 wt% (+19%), Zr1-r: ~43.6 wt% (+25%)). Conversely, UV treatment did not affect the crystalline structure of Zr2-m and Zr2-r (Figure 4A–D).

### 3.3. Hydrophilic Status of Different Surfaces

**Figure 5:**
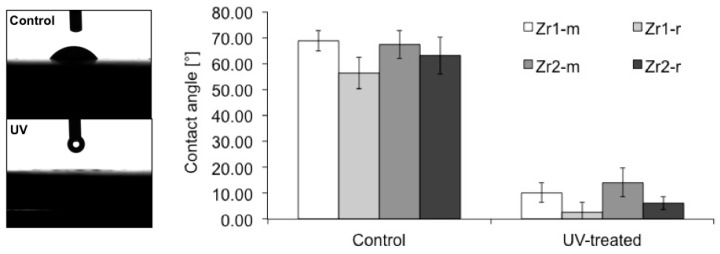
Alteration of the hydrophilic property of zirconia disks with smooth and roughened surfaces after UV treatment. A photographic image of 1-μL H_2_O droplets pipetted onto zirconia disks before and after the application of UV light (**left**). The histograms show the contact angle of 1-μL H_2_O droplets measured by an automatic contact angle measuring device before and after UV treatment on different zirconia disks with smooth and roughened surfaces (**right**).

The contact angle measurements of a 1-µL H_2_O droplet on untreated control zirconia-based disks were: Zr1-m, 68.8° ± 4.04°; Zr1-r, 56.4° ± 6.07°; Zr2-m, 67.4° ± 5.35°; and Zr2-r, 63° ± 7.1°, which indicated a low interaction with H_2_O molecules (Figure 5). After UV treatment was applied, the hydrophilic status of all samples changed significantly from hydrophobic to hydrophilic or even superhydrophilic (*p* < 0.0001) (Figure 5). The averaged contact angles of UV-treated samples were: Zr1-m, 10.25° ± 1.12°; Zr1-r, 2.5° ± 4.04°; Zr2-m, 14.08° ± 1.33°; and Zr2-r, 6.04° ± 1.72° (Figure 5).

### 3.4. Effect of UV Treatment on Surface Chemical Composition

**Figure 6:**
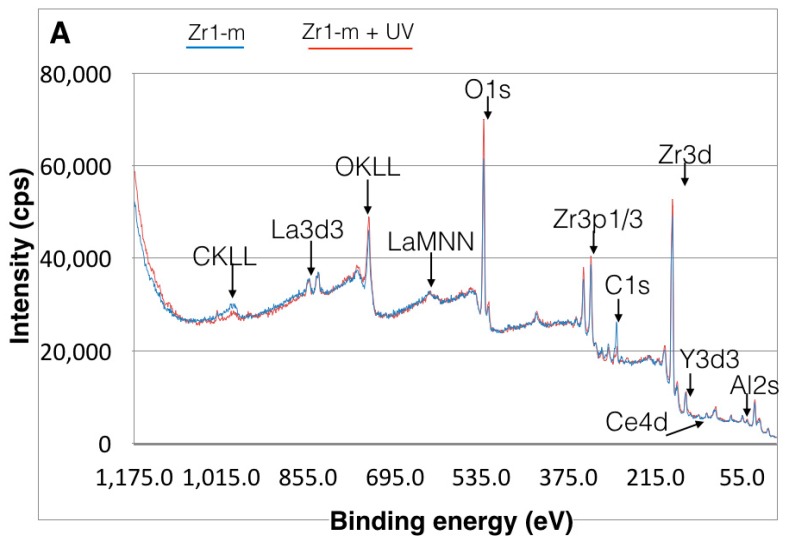

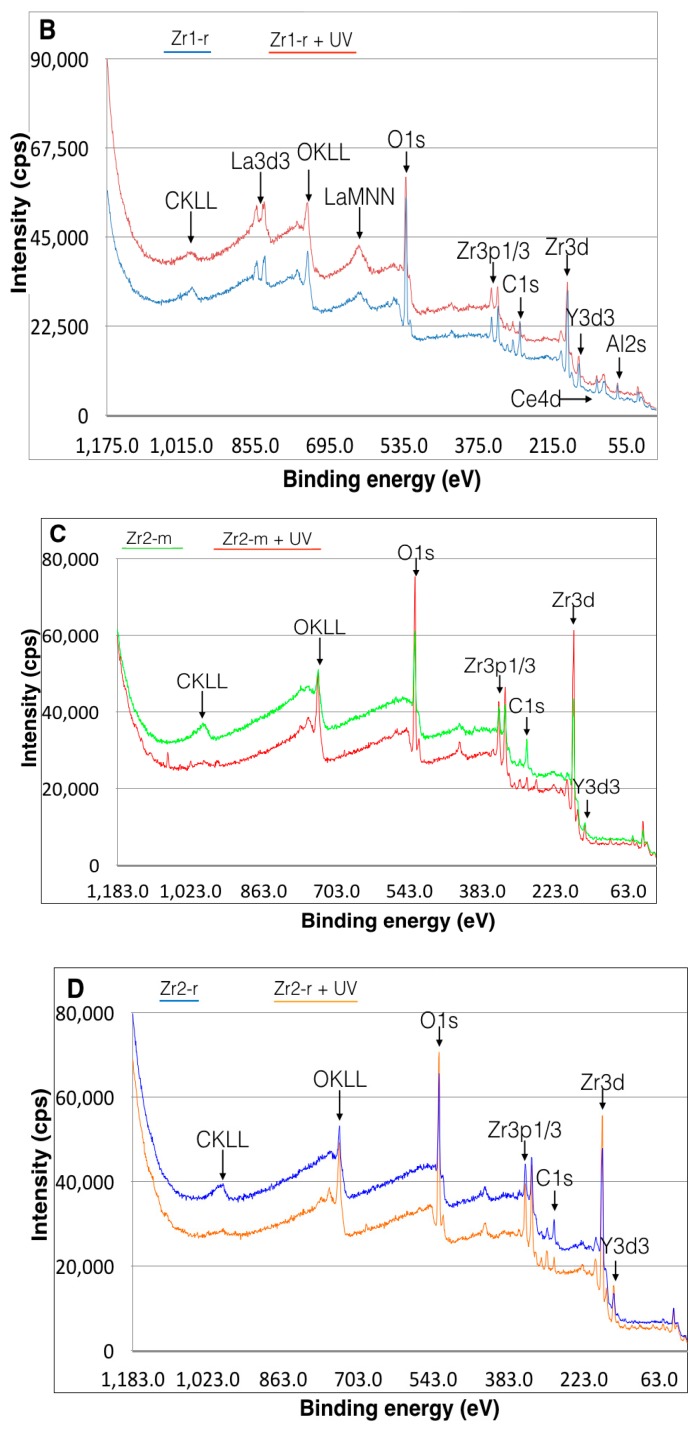
(**A**–**D**) X-ray photoelectron spectroscopy (XPS) spectrum for Zr1-m, Zr1-r, Zr2-m and Zr2-r surfaces before and after UV treatment.

**Figure 7:**
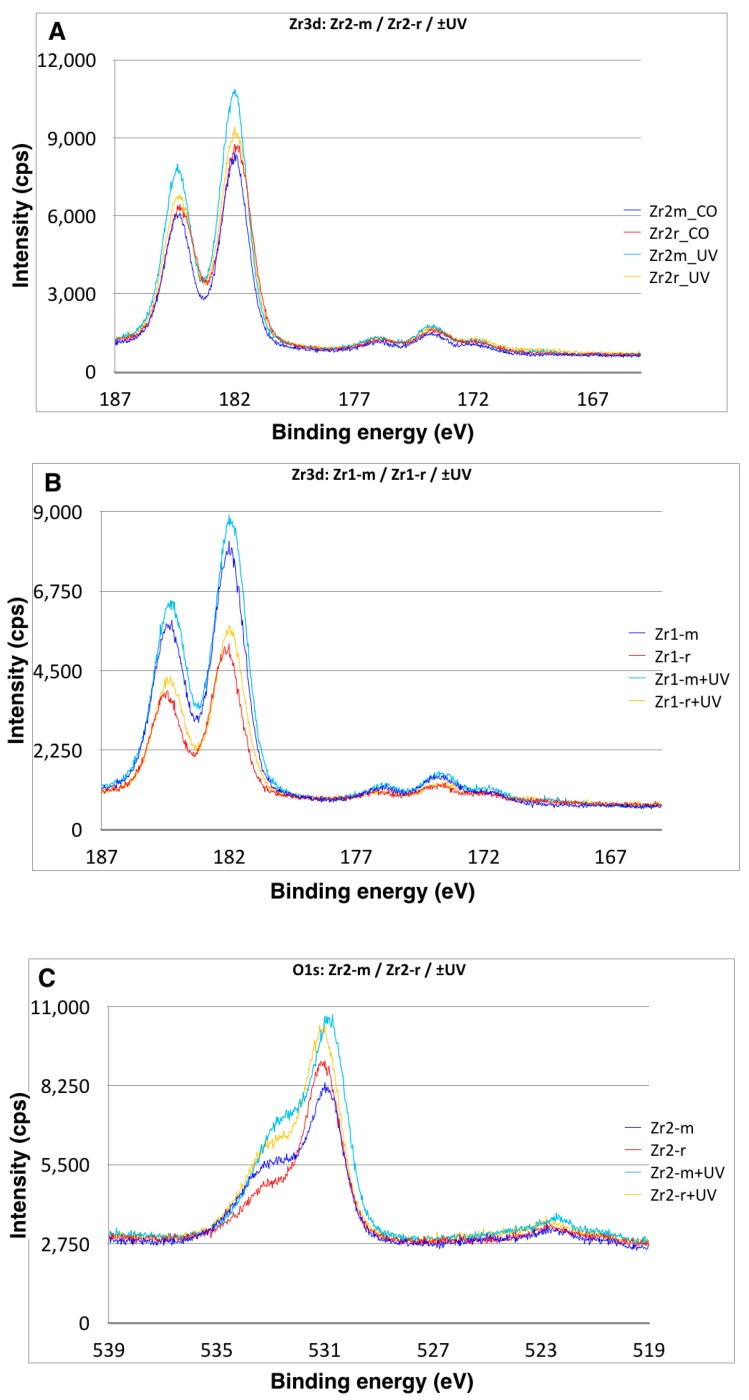

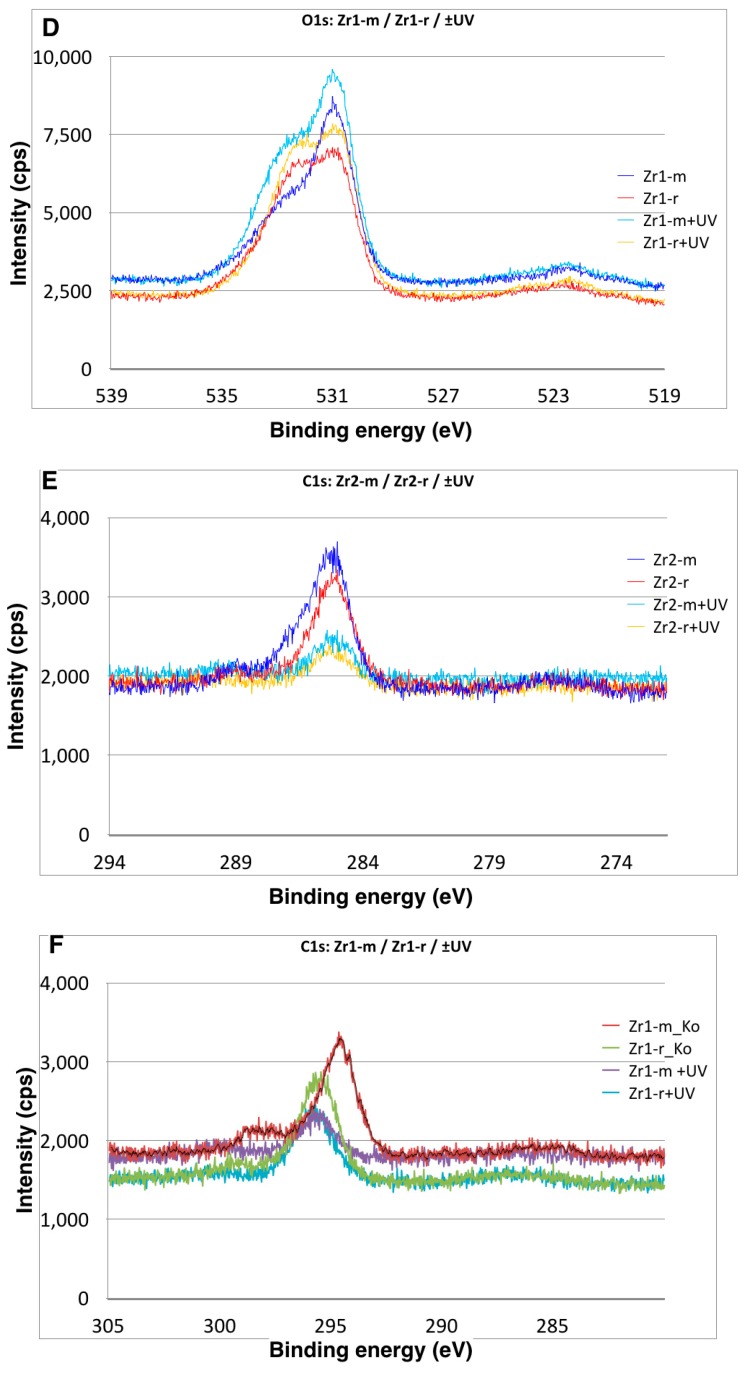
A close-up view of the XPS peaks for (**A**,**B**) Zr3d, (**C**,**D**) O1s and (**E**,**F**) C1s of the Zr1-m, Zr1-r, Zr2-m and Zr2-r surfaces, indicating changes in the atomic percentage of surface C, O and Zr after UV treatment.

The XPS spectra of all zirconia-based surfaces showed peaks of C1s, O1s, Zr3d3, Y3d3 and Hf4p1 (Figure 6A–D). Additional peaks were detected on material Zr1, including Al2p, La3d5 and Ce4d (Figure 6A–D). The Zr3d3 peak was located at about 185 eV, which can be assigned to the oxidized Zr4^+^ state (ZrO_2_) (Figure 6A–D). The binding energy of the Y3d3 peak at 157.9 eV indicated the presence of Y_2_O_3_. For material Zr1, the binding energy of the Al2p peak at 72.9 eV and La3d5 peak at 836.0 eV corresponds to Al_2_O_3_ and to La_2_O_3_, respectively (Figure 6A–D). A shoulder peak of C1s at around 291.0 eV can be ascribed to a carbonyl group containing hydrocarbons. The detailed spectra for the C1s electrons revealed a surface carbon amount of 28% on Zr2-r, 30% on Zr1-r, 34% on Zr1-m and 36% on Zr2-m (Figure 7A–F). Further XPS analysis for zirconia-based samples with UV treatment revealed that the C1s peak at 285 eV ascribed to oxygen-containing hydrocarbons decreased by 43%–81% on all surfaces, whereas an increase of Zr3d by 9%–41% and O1s by 19%–45% was observed (Table 2). The extent of change in the peak intensities of the electron energy levels was higher for smooth surfaces than for roughened surfaces (Table 2).

### 3.5. UV-Enhanced In Vivo Implant Fixation

**Figure 8:**
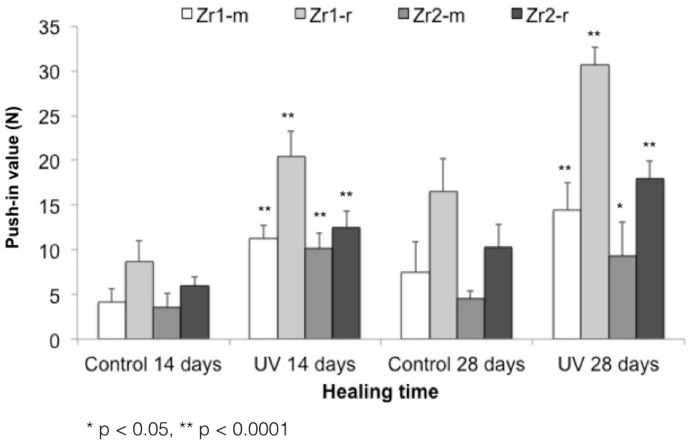
Push-in values around Zr1-m, Zr1-r, Zr2-m and Zr2-r before and after UV treatment.

The biomechanical push-in test values at Week 2 of healing yielded significantly stronger bone-zirconia integration for all UV-treated implants relative to untreated samples. The values were 2.1-, 2.3-, 2.8- and 2.1-fold greater (*p* < 0.05, *p* < 0.0001) for UV-treated Zr1-m, Zr1-r, Zr2-m and Zr2-r, respectively (Figure 8; push-in values around Zr1-m, Zr1-r, Zr2-m and Zr2-r before and after UV treatment). After four weeks of healing, the UV-treated implants still maintained their superiority over untreated samples, showing 1.9-, 1.8-, 2.04- and 1.7-fold greater push-in values for Zr1-m, Zr1-r, Zr2-m, and Zr2-r, respectively (Figure 8; push-in values around Zr1-m, Zr1-r, Zr2-m and Zr2-r before and after UV treatment).

### 3.6. Bone Morphogenesis around UV-Treated Implants

**Figure 9:**
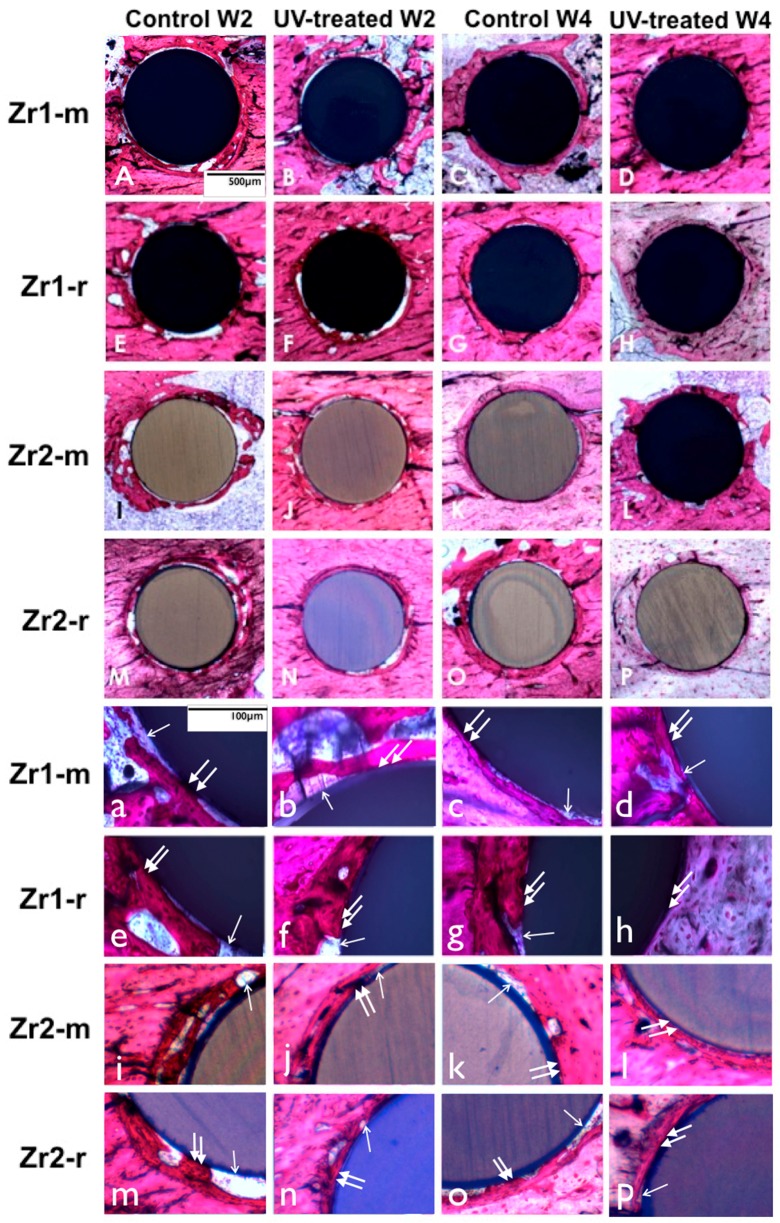
(**A**–**P**) UV light-promoted peri-implant bone generation at Weeks 2 and 4 of healing time. Representative histological images of the zirconia-based smooth and roughened implants with toluidine blue and Pararosanilin stain in an original magnification of 10× (**A**–**P**) and 40× (**a**–**p**). UV-treated implants are associated with a higher amount of bone-implant contact than non-treated implants. UV-treated implants at Week 2 (b,f,j,n) are associated with more vigorous bone formation with less interposition of soft tissue between the bone and the implant surface (arrowheads (b,f,j,n)); in contrast, the bone around untreated implants appears to be fragmentary (a,e,i,m) and involves more soft tissue (arrows (a,e,i,m)), interfering with the establishment of the direct bone implant contact; at Week 4, extensive bone spread can be observed along the implant surface without soft tissue interposition (arrowheads (d,h,l,p)) around UV-treated implants, whereas the bone around untreated implants is largely kept apart from the implant surface by soft tissue (arrows (c,g,k,o)).

At Week 2 of healing, bone tissue with a woven, immature appearance formed in an area relatively distant from the implant surfaces in both the control and UV-treated smooth and rough implants (Figure 9). The examination in the immediate vicinity of the implant surface revealed that bone formation was more extensive around UV-treated implants with less connective tissue interposition. Another notable difference was the greater amount of newly-formed bone around implants with a rougher surface configuration when compared to smooth surfaces. At Week 4 of healing, some parts of the untreated control implants (smooth and rough surfaces) still exhibited fibrous connective tissue intervening between bone and implant surface (Figure 9). However, the extent of soft tissue intervention seemed to be smaller than that observed at Week 2 (Figure 9). On the other hand, UV-treated implants exhibited almost no interposition of connective tissue between the implant surfaces and bone tissue. Implants with roughened surfaces were almost entirely surrounded by directly-deposited bone, in contrast to smooth surfaces, for which some areas with connective tissue were observed (Figure 9).

**Figure 10:**
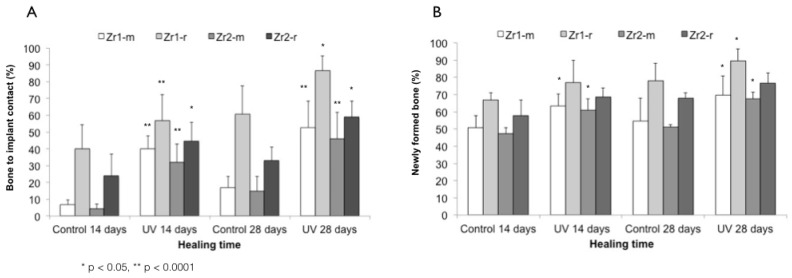
Histomorphometry values of bone-implant contact (**A**); bone volume (**B**) around Zr1-m, Zr1-r, Zr2-m and Zr2-r before and after UV treatment.

The histomorphometric evaluation of peri-implant bone revealed that UV treatment significantly increased the percentage of bone-implant contact around all samples (*p* < 0.0001 and *p* < 0.05) (Figure 10A). The enhancement was 3–7-fold for smooth surfaces and 1.4–1.7-fold for rough surfaces at Weeks 2 and 4. Bone histomorphometry of UV-treated implants at Week 2 revealed a BIC of 40.2%, 56.8%, 31.8% and 44.7% around Zr1-m, Zr1-r, Zr2-m and Zr2-r, respectively. A similar trend was observed at Week 4 of healing, with BIC of 52.7%, 86.5%, 45.9% and 58.9% around Zr1-m, Zr1-r, Zr2-m and Zr2-r, respectively (Figure 10A). Furthermore, UV treatment consistently increased the amount of bone volume in the area proximal to the implant surface by 20%–24% around smooth surfaces and by 11%–13% around roughened surfaces of both zirconia-based materials at Weeks 2 and 4 (*p* < 0.05) (Figure 10B).

## 4. Discussion

To our knowledge, this is the first report addressing the influence of UV treatment on the osteoconductive capacity of zirconia-based implant materials with different surface features. UV treatment substantially enhances the osteogenesis process, resulting in a greater amount of peri-implant bone, as well as an increased strength of bone-zirconia integration. In addition, the histomorphometric and push-in values of UV-treated implants at Week 2 reached similar or even higher values compared to non-treated implants at Week 4, indicating that UV treatment also accelerated the osseointegration process.

The term ‘photofunctionalization’ has previously been described as the effect of UV light on titanium surfaces. The change in the wettability behavior of oxides of Ti and Ti alloys upon UV light treatment was attributed to the electrostatic properties of TiO_2_ and its high photocatalytic activity [26,27,36,37]. Accordingly, UV irradiation induces a provisional chemical alteration within the superficial layer of TiO_2_, leading to a photocatalytic chemical reaction [38,39] by the excitement of electrons from the valence to the conduction band [39,40]. It has been suggested that the surface oxygen vacancies can be formed at bridging sites, resulting in the conversion of Ti^4+^ sites to Ti^3+^, which are favorable for dissociative water adsorption [25,39], inducing the surface wettability. Furthermore, this photocatalytic activity leads to a decomposition of native organic contaminants on the surface of TiO_2_ and transforms the negatively-charged superficial TiO_2_ layer to electropositive [37]. Additionally, UV treatment generates a decrease in the atomic percentage of surface hydrocarbon, whereas the amount of other elements uniformly increased according to the reduction of superficial hydrocarbons [36]. Based on the previous findings, the mechanism of UV photofunctionalization-induced titanium osteoconductivity is based on the increased wettability, the reduction of surface hydrocarbons and the change in the surface charge [26,36,41]. These findings are in line with several in vitro studies, which showed an increased cell attachment and proliferation, as well as protein adsorption on UV-treated titanium surfaces compared to the untreated samples, which explains the UV-induced osteoconduction of titanium [41,42].

Similar to titanium, UV application on zirconia has been shown to induce a decrease in the atomic percentage of carbon, whereas the amount of other elements uniformly increased according to the reduction of superficial hydrocarbons [26,27,41]. Hence, UV treatment of zirconia has been proposed to induce electron excitation from the valence band to the conduction band, provided the photon energy is sufficient [24]. Compared to titanium, zirconia requires photon energy larger than 5 eV to cause electrons to go from the V to C band to express its photocatalytic activity [28]. In regards to the surface properties, several reports have demonstrated the transformation of a zirconia surface from hydrophobic (contact angle, 100.1 degrees) to hydrophilic (contact angle, 20.8 degrees). This transformation was associated with the reduction of the atomic percentage of surface carbon in a dose-dependent manner from >50% down to <20% after UV treatment. Clearly, there is a negative correlation between the UV exposure dose and the percentage of surface carbon. The findings indicated that photocatalytic and photochemical degradation and the removal of hydrocarbons from the zirconia surface by UV treatment are similar to those seen on titanium surfaces.

On the other hand, the effect of UV photofunctionalization on the cell attractiveness of zirconia implant materials has been evaluated in several in vitro studies [24,28]. Primary human alveolar bone-derived osteoblasts depicted a significantly higher number of attached cells, a higher proliferation activity and increased mineralized nodules on the UV-treated samples compared to untreated control group [24]. Distinctly, the results of the different studies support the hypothesis that zirconia-based implant materials react to UV treatment in a similar manner to that of titanium. The results of the current study clearly demonstrated that UV photofunctionalization enhances the osteoconductive capacity of zirconia-based implant materials. Although it can be proposed that this enhancement is similar to that of titanium, the mechanism of increased osteoconductivity of zirconia upon UV light treatment is yet to be clarified.

These observations are in line with previous reports using other materials: in a similar experimental set-up; UV treatment of micro-roughened titanium implants placed in rat femurs enhanced bone response and resulted in a faster and stronger osteogenesis [26,27]. The bone-titanium interface was three-times stronger for the UV-treated implants at Week 2 of healing, as demonstrated by the push-in test evaluation. Furthermore, histologic comparison showed that the bone-implant contact was maximized up to 100% at Week 4 around UV-treated implants, whereas untreated implants remained at 50% [26]. Additional osteomorphogenic distinctions, such as the amount of soft tissue interposition, were more pronounced around untreated implants when compared to UV-treated samples, where almost no soft tissue intervention was observed at Week 4 of healing [26]. In another study, acid-etched titanium implants of different lengths were compared with and without UV treatment [29]. The results showed that the mean push-in value of untreated standard-length implants was significantly higher than that of the untreated short (40% shorter than standard length) implants [29]. Surprisingly, the mean push-in value for UV-treated short implants was twice that of untreated standard-length implants at Week 2 of healing [29]. The similar results between previous reports and the current study indicate that UV treatment of zirconia surfaces enhances their osteoconductive capacity in a similar manner as for titanium.

The remarkable increase of bone morphogenesis around UV-treated surfaces, also defined as “super osseointegration”, has been ascribed to the amplified cell response and activity on UV-treated surfaces [36]. In fact, it has been reported that osteoblast response to titanium or zirconia can be enhanced by UV treatment, resulting in accelerated and augmented cell attachment, accelerated cell spread and cytoskeletal development, as well as increased proliferation [26,28,29]. The strong cellular response to UV-treated surfaces has been previously associated with an increase in surface hydrophilicity [26,28,29]. In this study, UV treatment converted the hydrophilic status of zirconia surfaces from hydrophobic to superhydrophilic (contact angle <5°). However, evidence directly linking the increased surface hydrophilicity and its effect on improved bone formation is still controversial. According to earlier research, it is not a universally-accepted principle that the hydrophilicity of a material’s surface is proportional to its bioactivity [43,44,45,46]. A series of previous studies demonstrated that maintaining the hydrophilic properties of titanium surfaces appears to have a positive effect on increasing the attachment, spread and proliferation of osteogenic cells. Nevertheless, mechanistic evidence indicating that hydrophilicity of a material is critical in determining its bioactivity or osteoconductivity is still lacking [26,28,32,33,41]. On the other hand, the level of surface hydrocarbon has been strongly correlated with the implant surface’s capacity for protein adsorption and cell attachment [26]. When the amount of carbon-containing compounds on the surface decreased, the biologic capacity of zirconia and titanium increased, resulting in the amplification of the osteoblast response and greater bone-to-implant contact [26,28]. Mechanisms linking the removal of hydrocarbon and the increased biomaterial osteoconductivity can be ascribed to the change in the electrostatic potential of the implant surface [26]. In this study, the extent to which the osteoconduction is enhanced seems to be different between the two tested materials. This may be attributed to the different surface characteristics of zirconia samples, as well as the difference in the chemical composition of both materials and their UV absorption capacity. The possible effect of additional elements on the UV effect cannot be excluded and might be the cause of the osseointegration differences, as the zirconia samples of the material Zr1 contained less ZrO_2_ (85.7 wt%) and Y_2_O_3_ (4.3 wt%), but more Al_2_O_3_ (8.3 wt%) and even additional La_2_O_3_ (1.7 wt%) compared to Zr2 (93 wt% ZrO_2_, 5 wt% Y_2_O_3_, 1.9 wt% HfO_2_ and 0.1 wt% Al_2_O_3_) [24,25].

Recent studies have revealed that new titanium surfaces are electropositively charged immediately after processing and lose this status over time as hydrocarbons accumulate on the surface [32,41]. This progressive accumulation of carbonyl-containing compounds is considered unavoidable under ambient conditions and produces an electronegatively-charged surface, which is no longer protein attractive, leading to a decrease in protein adsorption [37,41]. Exposing such titanium surfaces to UV light removes the oxygen-containing hydrocarbons and creates Ti^4+^ sites, which restores the electropositive surface charge [47]. This allows for a 30%–100% increase in adsorption of negatively-charged proteins, such as albumin and fibronectin, on titanium surfaces [32,41,48]. As a result of increased protein adhesion, subsequent cell attachment is enhanced in correlation with an increased cell-protein interaction via ligand-specific binding (e.g., integrin–RGD interaction) [41,48]. Further studies are needed to elucidate the mechanism by which UV treatment enhances the bioactivity of implant surfaces on the protein and cellular levels.

To determine whether the observed increase in the osteoconductive capacity found in our study can be ascribed to the above-mentioned UV-induced effects, we carefully characterized the zirconia-based samples. Based on the XPS analysis, UV treatment of the zirconia-based samples led to considerable chemical alterations, which were characterized by a significant reduction of surface carbons by 50%–75% on both rough and smooth surfaces. At the same time, the percentage of other elements, such as oxygen and Zr^4+^, uniformly increased. The histomorphometric analysis, as well as the biomechanical evaluation, showed a faster bone formation process with a considerably greater amount of newly-formed bone around UV-treated implants, when compared to non-treated specimens. Furthermore, UV-irradiated implants were also correlated with higher push-in values, indicating enhanced bone-zirconia integration. Based on these findings, we hypothesize that similar physicochemical changes, as previously described for titanium, also occur on zirconia-based surfaces after UV treatment and that a similar biological process between proteins and cells is responsible for the observed increase in the biomaterial osteoconductive capacity. Further investigations are needed to verify the mechanism of UV treatment-generated osteoconductivity, as well as the possible adverse effects of UV irradiation on the mechanical and water absorbing properties and the discoloration of zirconia. The present results may open a new path in the surface modification of zirconia implants and provide an important insight into further advancing the research on exploring the impact of this technique on the degree of osseointegration in a human trial.

## 5. Conclusions

UV treatment enhanced the osteoconductivity of zirconia-based materials with different surface characteristics, resulting in an augmented amount of bone formation and the more rapid establishment of bone-implant integration. Further studies are needed to verify these findings in a human setting.

## Figures and Tables

**Table 1:** Surface roughness values of different groups measured by AFM. No significant changes were observed in Ra, Rz or Rms values after UV treatment of zirconia samples.

Sample	Ra (µm)		Rms (µm)		Rz (µm)	
	Control	UV	Control	UV	Control	UV
**Zr1-m**	0.12 (±0.02)	0.09 (±0.02)	0.17 (±0.03)	0.13 (±0.02)	1.04 (±0.21)	0.76 (±0.09)
**Zr1-r**	0.21 (±0.08)	0.29 (±0.07)	0.29 (±0.07)	0.36 (±0.08)	1.3 (±0.18)	1.39 (±0.2)
**Zr2-m**	0.03 (±0.01)	0.03 (±0.01)	0.04 (±0.01)	0.04 (±0.01)	0.20 (±0.02)	0.19 (±0.03)
**Zr2-r**	0.31 (±0.11)	0.27 (±0.03)	0.38 (±0.13)	0.34 (±0.04)	1.67 (±0.26)	1.53 (±0.11)

**Table 2:** The detailed XPS spectra of C1s, O1s and Zr3d show that UV-light treatment decreased the atomic percentage of carbon and increased the amount of oxygen and Zr3d on smooth and roughened surfaces.

	C1s (at%) *	C1s + UV (at%)	O1s (at%)	O1s + UV (at%)	Zr3d (at%)	Zr3d + UV (at%)
**Zr1-m**	33	07 (77%)	48	70 (45%)	19	23 (22%)
**Zr1-r**	31	18 (43%)	57	68 (19%)	13	15 (17%)
**Zr2-m**	37	08 (81%)	47	68 (44%)	16	23 (41%)
**Zr2-r**	27	07 (71%)	52	69 (33%)	23	25 (09%)

* (%) The presented values indicate the difference between the percentage of each element on the surface before and after UV.
